# The effect of propofol on hypoxia‐ and TNF‐α‐mediated BDNF/TrkB pathway dysregulation in primary rat hippocampal neurons

**DOI:** 10.1111/cns.13809

**Published:** 2022-02-03

**Authors:** Weiping Tao, Xuesong Zhang, Juan Ding, Shijian Yu, Peiqing Ge, Jingfeng Han, Xing Luo, Wei Cui, Jiawei Chen

**Affiliations:** ^1^ Department of Anesthesiology Jing’an District Central Hospital of Shanghai Shanghai China; ^2^ Department of Anesthesiology Shanghai Public Health Clinical Center Shanghai China; ^3^ Department of Anesthesiology Department of Oncology Fudan University Shanghai Cancer Center Shanghai Medical College Fudan University Shanghai China

**Keywords:** Brain‐derived neurotrophic factor, Hippocampal neuron, Hypoxia, Propofol, Tumor necrosis factor‐α

## Abstract

**Aims:**

Hypoxia and inflammation may lead to BDNF/TrkB dysregulation and neurological disorders. Propofol is an anesthetic with neuroprotective properties. We wondered whether and how propofol affected BDNF/TrkB pathway in hippocampal neurons and astrocytes.

**Methods:**

Primary rat hippocampal neurons and astrocytes were cultured and exposed to propofol followed by hypoxia or TNF‐α treatment. The expression of BDNF and the expression/truncation/phosphorylation of TrkB were measured. The underlying mechanisms were investigated.

**Results:**

Hypoxia and TNF‐α reduced the expression of BDNF, which was reversed by pretreatment of 25 μM propofol in hippocampal neurons. Furthermore, hypoxia and TNF‐α increased the phosphorylation of ERK and phosphorylation of CREB at Ser142, while reduced the phosphorylation of CREB at Ser133, which were all reversed by 25 μM propofol and 10 μM ERK inhibitor. In addition, hypoxia or TNF‐α did not affect TrkB expression, truncation, or phosphorylation in hippocampal neurons and astrocytes. However, in hippocampal neurons, 50 μM propofol induced TrkB phosphorylation, which may be mediated by p35 expression and Cdk5 activation, as suggested by the data showing that blockade of p35 or Cdk5 expression mitigated propofol‐induced TrkB phosphorylation.

**Conclusions:**

Propofol modulated BDNF/TrkB pathway in hippocampal neurons via ERK/CREB and p35/Cdk5 under the condition of hypoxia or TNF‐α exposure.

## Graphical abstract

In rat hippocampal neurons, propofol via modulating ERK/CREB signaling pathway may reverse hypoxia‐ and TNF‐α‐mediated reduction in BDNF. In hippocampal neurons, propofol via activating p35/Cdk5 pathway may induce TrkB phosphorylation. These effects are correlated with hippocampal neuron viability.

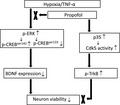

## 1 INTRODUCTION

Brain‐derived neurotrophic factor (BDNF) is one of the most studied and well‐characterized neurotrophic factors in central nervous system (CNS), and is mainly produced in the brain by hippocampal neurons and astrocytes. BDNF plays an extensive role in neuronal growth, differentiation, survival, synaptic plasticity, and neurotransmitter regulation.^1^ As such, BDNF may have therapeutic potential for the treatment of neurological disorders, such as cerebral ischemia‐reperfusion injury,^2^ neuroinflammation‐related brain injury,^3^ age‐related memory impairment,^4^ Parkinson’s disease,^5^ Alzheimer’s disease,^6^ and postoperative cognitive dysfunction.^7^ It was originally thought that BDNF exerts its biological functions through binding to two transmembrane receptors, the tropomyosin receptor tyrosine kinase B (TrkB) and p75 neurotrophin receptor (p75NTR). However, accumulating evidence suggested that mature BDNF has high affinity to TrkB, while its precursor proBDNF mainly activates p75NTR.^8^

Numerous *in vitro* and animal studies have implied a role for BDNF/TrkB in CNS damages induced by multiple pathophysiological stimuli such as oxidative stress, inflammation, and ischemia/reperfusion injury.^9^ Recently, BDNF/TrkB signaling has been identified as a potential therapeutic target for depression,^10^ post‐cerebral ischemic spatial cognitive dysfunction,^11^ vascular dementia,^12^ and postoperative cognitive dysfunction.^13^

Propofol is (2, 6‐diisopropyl phenol) an intravenous general anesthetic used extensively in the induction and maintenance of anesthetization and procedural sedation. Apart from its multiple anesthetic advantages, it has been reported to possess anti‐oxidative and anti‐inflammatory effect^14^, ^15^ as well as neuroprotective property.^16^ A number of *in vitro* studies revealed that propofol may protect mouse hippocampal neurons from autophagy induced by inflammation^17^ and from apoptosis induced by inflammation, hypoxia, or oxidative stress.^18^, ^19^, ^20^ In addition, *in vitro* models indicated propofol may protect the integrity of blood‐brain barrier (BBB) from impairment by hypoxia and inflammation.^17^, ^21^, ^22^, ^23^ Although propofol has been reported to modulate the expression of BDNF and TrkB in the hippocampus of cerebral ischemia injury^24^ in aged rats, the effects of propofol on BDNF/TrkB pathway in the neurons have not been thoroughly investigated.

Although multiple pathological factors may affect CNS, it is acknowledged that CNS is especially vulnerable to ischemia and inflammation.^25^ Furthermore, in vitro models proved that hypoxia treatment and inflammation cytokine TNF‐α treatment may induce neuron injury, and mimic in vivo ischemic or inflammatory status.^26^, ^27^ In the current study, we aimed to detect whether propofol could modulate hypoxia‐ and TNF‐α‐mediated BDNF/TrkB pathway dysregulation in primary rat hippocampal neurons and astrocytes, and further investigate the underlying mechanisms. Our findings may provide a novel therapeutic target for hypoxia‐ and inflammation‐induced neuron injury.

## 2 MATERIALS AND METHODS

### 2.1 Experimental design

Primary rat hippocampal neurons and astrocytes were cultured in normoxic condition (95% humidified air and 5% CO_2_) until ready for experiments. To mimic hypoxic condition, cells were maintained in a hypoxic chamber flushed with a humidified gas mixture (90% humidified N_2_, 5% O_2_, and 5% CO_2_) for different duration (0, 1, 2, 3, 6, and 12h). To mimic inflammation condition, cells were treated with 40ng/mL TNF‐α for different duration (0, 1, 2, 3, 6, and 12h). To examine the effect of propofol, cells were treated with different concentrations (1, 5, 10, 25, 50, and 100 μM) of propofol (Sigma‐Aldrich, St. Louis, MO, USA) or its solvent, 0.1% dimethyl sulfoxide (DMSO) for 1h, and then exposed to hypoxic or inflammation condition. These treatment conditions have been repeatedly used to induce vicious effects in hippocampal neurons.^17^, ^21^ We intended to identify the effect of hypoxia, inflammation, and propofol on the production of BDNF and the expression/truncation/phosphorylation of TrkB in hippocampal neurons and astrocytes. More importantly, we aimed to investigate the mechanisms, including extracellular regulated protein kinase (ERK), cAMP response element‐binding protein (CREB), p35, and cyclin‐dependent kinase 5 (Cdk5). To confirm their roles, specific inhibitors and short‐interference RNAs (siRNAs) were applied.

### 2.2 Cell culture

Primary rat hippocampal neurons and astrocytes were purchased from ScienCell Research Laboratories (Carlsbad, CA, USA). The cryopreserved primary rat hippocampal neurons were thawed and seeded into tissue culture flasks containing 5 mL neuronal medium, which was supplemented with neuronal growth supplement and 1% penicillin/streptomycin. The culture media were replaced every 2–3 days. Neurons were incubated at 37°C in a humidified atmosphere with 5% CO_2_, and were ready for experiments without sub‐culturing.

The cryopreserved rat astrocytes were thawed and seeded into tissue culture flasks containing 5ml astrocyte medium‐animal, which was supplemented with 2% fetal bovine serum (FBS), astrocyte growth supplement‐animal, and 1% penicillin/streptomycin. Astrocytes were incubated at 37°C in a humidified atmosphere with 5% CO_2_, and culture media were replaced every 2–3 days. The cells were sub‐cultured when reaching 80–90% confluency, and the fourth passage of astrocytes was used in the present study.

### 2.3 Cell viability assay

To check the effect of hypoxia, TNF‐α, and propofol on the viability of hippocampal neurons and astrocytes, 3‐(4,5)‐dimethylthiahiazo (‐z‐y1)‐3,5‐di‐phenytetrazoliumromide (MTT) protocol was used. In brief, cells were cultured in six‐well tissue culture plates until reaching 80% confluency. After respective treatment, the media were removed and the cells were carefully rinsed with phosphate‐buffered saline (PBS). Then, 5 mg/mL MTT was added to each well and incubated for 4h. Finally, the resulting formazan crystals were dissolved in DMSO, and the absorbance was measured by a spectrophotometer at 490 nm. Cell viability was expressed as the percentage of absorbance of treated cells compared with that of untreated control cells.

### 2.4 Protein preparation and measurement by Western blot analysis

For cellular protein isolation, hippocampal neurons and astrocytes were washed with PBS and scraped off the culture flasks. After centrifugation for 5 min at 1000 revolutions per minute (rpm), cell pellets were suspended in RIPA lysis buffer containing 1% protease inhibitor and 0.1% phosphatase inhibitor for 5 min. The proteins were obtained by centrifuging for 5 min at 3000 rpm, and quantified by BCA Assay kit (Beyotime Institute of Biotechnology, Shanghai, China).

Equal amounts of protein (about 60 μg) were separated via 8% or 10% SDS‐PAGE and electrophoretically transferred to polyvinylidinene fluoride membranes (Millipore Sigma, Shanghai, China). Following blocking in 5% skimmed milk at room temperature for 2h, the membranes were incubated overnight at 4°C with the following primary antibodies purchased from Cell Signaling Technology (MA, USA): anti‐ BDNF, anti‐ERK, anti‐phosphorylated ERK, anti‐CREB, anti‐ phosphorylated CREB^Ser142^, anti‐phosphorylated CREB^Ser133^, anti‐full‐length TrkB, anti‐truncated TrkB, anti‐phosphorylated TrkB, anti‐p35, anti‐p39, anti‐Cdk5, and anti‐GAPDH. Subsequently, the membranes were washed and incubated with corresponding HRP‐conjugated secondary antibody (Santa Cruz Biotechnology, CA, USA) at room temperature for 2h. Protein bands were visualized with Amersham ECL plus Western blotting detection reagent (Santa Cruz Biotechnology, CA, USA), and semi‐quantified with Image J v1.8.0 software.

### 2.5 mRNA analysis by RT‐PCR

After treatment, total RNA was isolated from cells with the use of RNeasy Mini kit (Qiagen, Hilden, Germany) according to the manufacturer’s instructions. Equal amount of total RNA (3 µg) was reverse transcribed into cDNA with oligo‐dT and M‐MLV reverse transcriptase at 42°C for 1h. Two microliter of reverse transcription (RT) material was amplified with Taq DNA polymerase and a primer pair specific to BDNF or GAPDH. For BDNF, forward primer 5’‐ AGC GCG AAT GTG TTA GTG GT‐3’and reverse primer 5’‐GCA ATT GTT GCC TCT TTT CT‐3’ were used; for GAPDH, forward primer 5’‐GAT GCT GGT GCT GAG TAT GRC‐3’ and reverse primer 5’‐GTG GTG CAG GAT GCA TTG CTC TGA‐3’ were used. The polymerase chain reaction (PCR) products were visualized in 1.5% agarose gel containing ethidium bromide. The intensity of BDNF bands was normalized with those of GAPDH bands, and the results were analyzed with Scan‐gel‐it software.

### 2.6 Transient transfection of siRNA

In this in vitro study, we used siRNA technology to knockdown specific gene expression. Cdk5 siRNA (sc‐29263), p35 siRNA (sc‐36154), p39 siRNA (sc‐42157), and control siRNA (sc‐37007) were purchased from Santa Cruz Biotechnology (CA, USA). siRNAs were delivered to hippocampal neurons using siRNA transfection reagent (sc‐29528, Santa Cruz Biotechnology, CA, USA) according to the manufacturer’s instructions. Briefly, for each transfection, dilute 5 µL siRNA duplex (50 pmol siRNA) into 100 µL siRNA transfection medium to obtain solution A, and dilute 5 µL siRNA transfection reagent into 100 µL siRNA transfection medium to obtain solution B. Mix solution A and solution B gently and incubate the transfection reagent mixture for 30 min at room temperature. Hippocampal neurons were seeded in six‐well tissue culture plates and cultured till reaching 60–70% confluence. Wash the neurons with siRNA transfection medium, overlay 0.5 mL transfection mixture onto the washed neurons, and incubate the neurons for 6h in 37°C incubator. Then, remove the transfection mixture, replace with normal growth medium, and incubate the neurons for an additional 18h in 37°C incubator. Thereafter, neurons were ready for experiments.

### 2.7 Measurement of Cdk5 kinase activity

Cdk5 kinase activity was analyzed by fluorescence assay using commercially available kits (Weike Biological Technology Company, Shanghai, China) according to the manufacturer’s instructions.

In brief, hippocampal neurons were seeded in 96‐well plates and subject to respective treatment. Then, neurons were washed and incubated with 200 μL Cdk5 kinase substrates solution supplemented with 5 mM MgCl_2_ and 0.5 mM ATP for 2h at 37°C. Cell culture plates were subject to a Clariostar TM spectrofluorimeter, and fluorescence emission was recorded at 680 nm following excitation at 620 nm. Cdk5 kinase fluorescence was calculated by subtraction of fluorescence from the values obtained in the absence of Cdk5 kinase. Experiments were performed in triplicate, and data were expressed as percentage of relative fluorescence compared with that of untreated control neurons.

### 2.8 Statistical analysis

Data were presented as mean ± standard deviation. All experiments were conducted with five independent repeats, which were performed with different cultures. The Shapiro‐Wilk test was used to analyze the normality of the data and we found that all data were normally distributed. Differences between groups were assessed with paired, two‐tailed Student’s *t*‐test or one‐way ANOVA, followed by post hoc Tukey testing. All statistical analyses were performed with SPSS software 11.5, and a significant difference was set at *P* < 0.05.

## 3 RESULTS

### 3.1 Hypoxia and TNF‐α reduced BDNF expression and cell viability in rat hippocampal neurons and astrocytes

**FIGURE 1:**
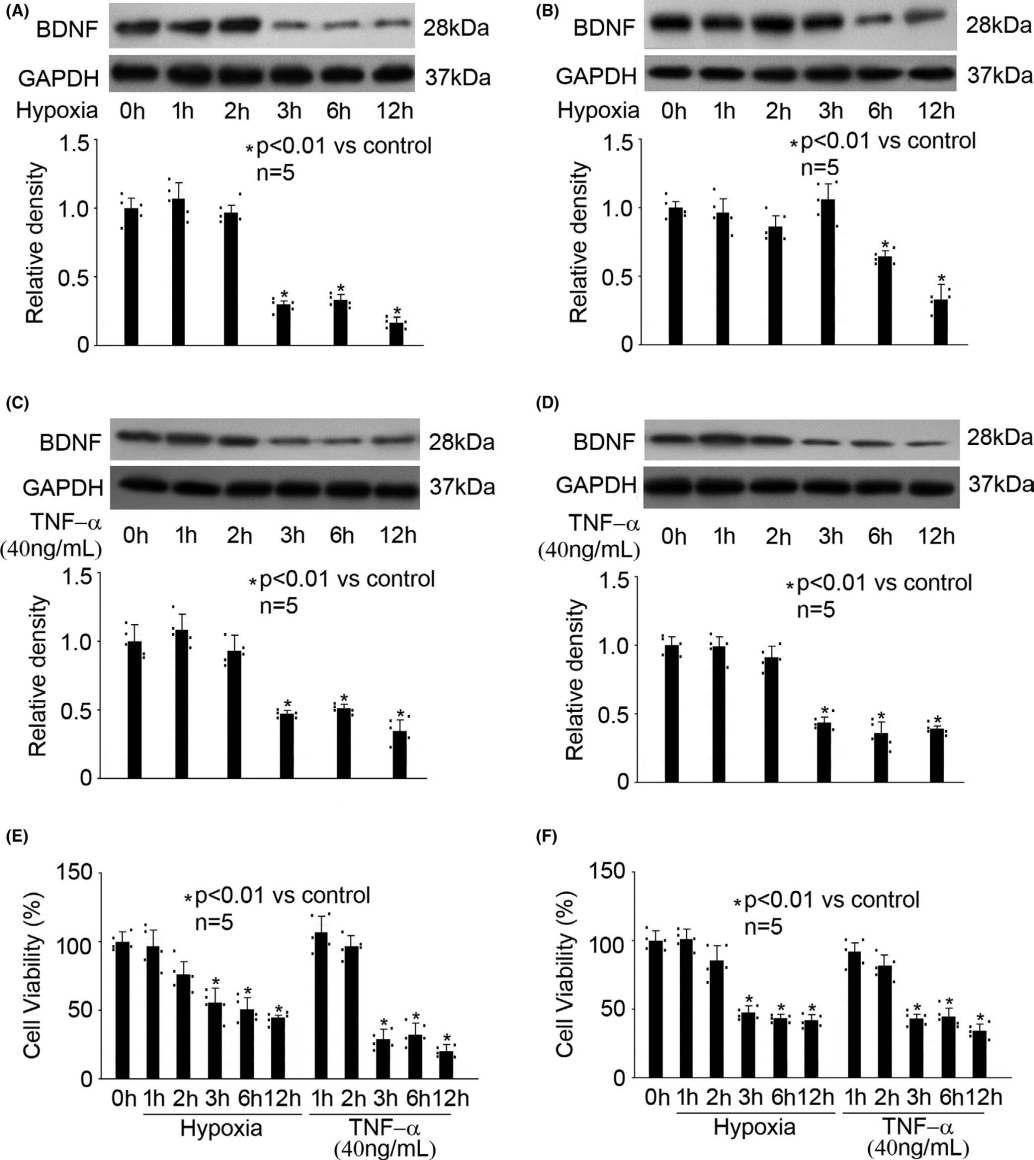
Hypoxia and TNF‐α reduced the expression of BDNF and cell viability in hippocampal neurons and astrocytes. The upper panel was a representative experiment and the lower panel was the summary of densitometric data from five separate experiments. GAPDH served as loading control. Data were expressed as normalized ratio of protein band density of BDNF against GAPDH, and were presented as mean ± standard deviation. Hypoxia treatment for 0h was considered as normoxic condition and served as control. (A) In hippocampal neurons, hypoxia reduced BDNF expression in a time‐dependent manner. (B) In astrocytes, hypoxia reduced BDNF expression in a time‐dependent manner. (C) In hippocampal neurons, TNF‐α reduced BDNF expression in a time‐dependent manner. (D) In astrocytes, TNF‐α reduced BDNF expression in a time‐dependent manner. (E) In hippocampal neurons, hypoxia and TNF‐α reduced cell viability in a time‐dependent manner. Data were expressed as the percentage of absorbance of treated cells compared with that of untreated control cells. (F) In astrocytes, hypoxia and TNF‐α reduced cell viability in a time‐dependent manner

Rat hippocampal neurons and astrocytes were cultured and exposed to hypoxia (5% O_2_) or TNF‐α (40 ng/mL) treatment for different duration (0, 1, 2, 3, 6, and 12h), and the expression of BDNF as well as cell viability was measured. As shown in Figure 1, we reported that in hippocampal neurons and astrocytes, hypoxia reduced BDNF protein expression in a time‐dependent manner, with the significant effects appearing at 3h in hippocampal neurons (Figure 1A, *P* < 0.01 vs. control) and at 6h in astrocytes (Figure 1B, *P* < 0.01 vs. control). In addition, we found that TNF‐α also reduced BDNF protein expression in a time‐dependent manner, and the significant effects appeared at 3h in both hippocampal neurons and astrocytes (Figure 1C,D, *P* < 0.01 vs. control). Furthermore, the effect of hypoxia and TNF‐α on BDNF protein expression was confirmed by measuring BDNF mRNA levels (Figure S1). We also showed that hypoxia and TNF‐α treatment reduced cell viability in hippocampal neurons (Figure 1E) and in astrocytes (Figure 1F), and the significant effects appeared after 3h treatment. Thereafter, these treatment conditions were applied in the following experiments to study the potential mechanisms.

### 3.2 Propofol reversed hypoxia‐ and TNF‐α‐modulated BDNF reduction and cell viability in rat hippocampal neurons

**FIGURE 2:**
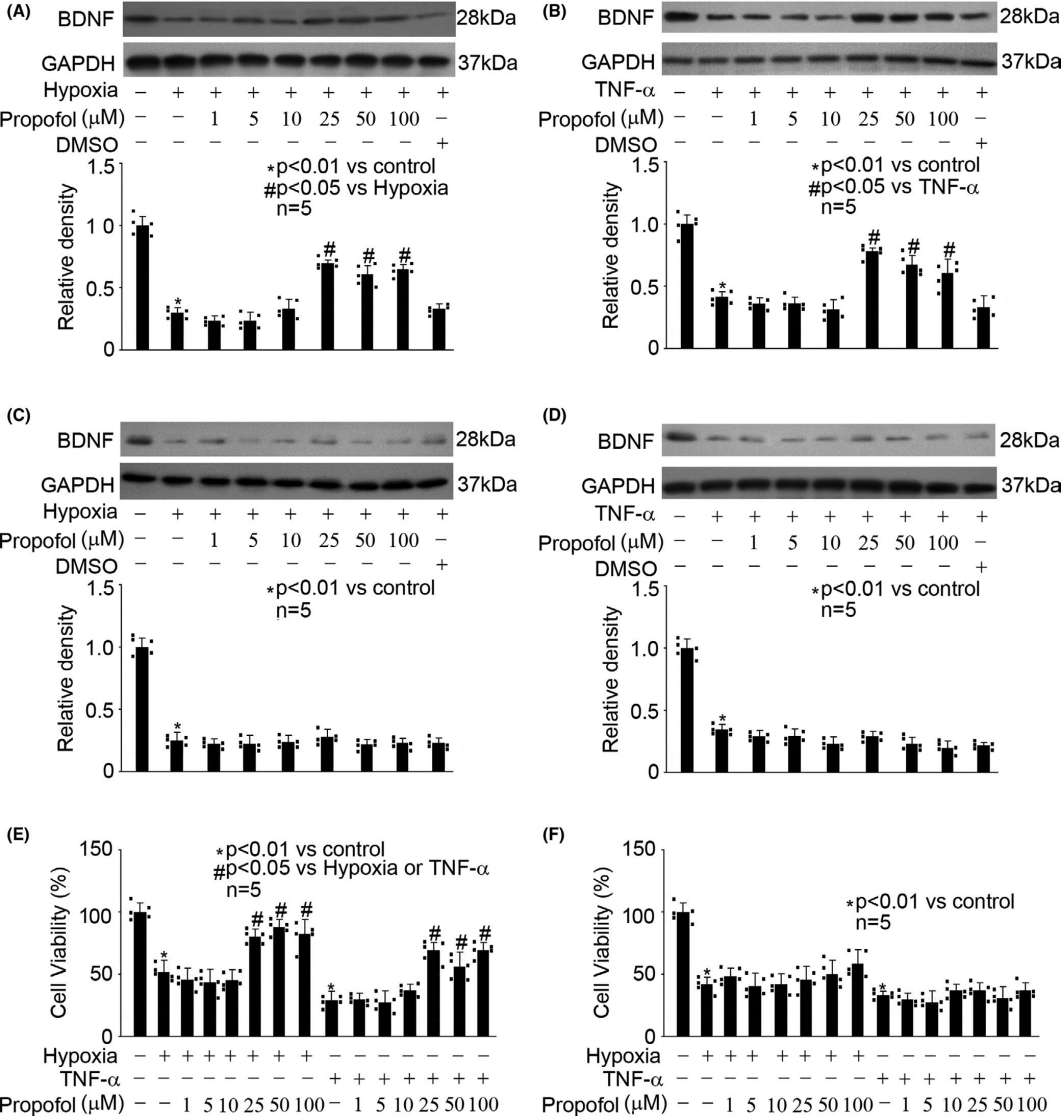
Propofol reversed hypoxia‐ and TNF‐α‐modulated BDNF reduction and cell viability in hippocampal neurons. The upper panel was a representative experiment and the lower panel was the summary of densitometric data from five separate experiments. GAPDH served as loading control. Data were expressed as normalized ratio of protein band density of BDNF against GAPDH, and were presented as mean ± standard deviation. (A) In hippocampal neurons, propofol induced BDNF expression, which was inhibited by hypoxia (5% O_2_, 3h) treatment. (B) In hippocampal neurons, propofol induced BDNF expression, which was inhibited by TNF‐α (40 ng/mL, 3h) treatment. (C) In astrocytes, propofol had no effect on hypoxia‐modulated BDNF expression. (D) In astrocytes, propofol had no effect on TNF‐α‐modulated BDNF expression. (E) In hippocampal neurons, propofol rescued cell viability, which was reduced by hypoxia or TNF‐α treatment. Data were expressed as the percentage of absorbance of treated cells compared with that of untreated control cells. (F) In astrocytes, propofol had no effect on hypoxia‐ or TNF‐α‐mediated cell viability

To observe the effects of propofol on hypoxia‐ and TNF‐α‐ modulated BDNF reduction and cell viability in hippocampal neurons and astrocytes, we pretreated cells with different concentrations of propofol (1, 5, 10, 25, 50, and 100 μM) for 1h, followed by hypoxia or TNF‐α treatment. As shown in Figure 2, in hippocampal neurons, propofol (25, 50, and 100 μM) induced BDNF protein expression, which was inhibited by hypoxia (5% O_2_, 3h) treatment (Figure 2A, *P* < 0.01 vs. control, *P* < 0.05 vs. hypoxia). Propofol (25, 50, and 100 μM) also induced BDNF protein expression, which was inhibited by TNF‐α (40 ng/mL, 3h) treatment (Figure 2B, *P* < 0.01 vs. control, *P* < 0.05 vs. TNF‐α). In contrast, we found that even 100 μM propofol had no or minor effect on BDNF protein expression in astrocytes in response to hypoxia or TNF‐α (Figure 2C,D). Furthermore, the effect of propofol on hypoxia‐ and TNF‐α‐modulated BDNF protein expression was confirmed by measuring BDNF mRNA levels (Figure S2). Meanwhile, our data indicated that propofol (25, 50, and 100 μM) rescued cell viability in hippocampal neurons (Figure 2E), but not in astrocytes (Figure 2F). Also, please note that 0.1% DMSO, the solvent for propofol, had no effect on BDNF protein production in hippocampal neurons or astrocytes (Figure 2). Therefore, we ruled out the role of DMSO. More importantly, we inferred that 25 μM propofol might be the minimally effective concentration that reversed hypoxia‐ and TNF‐α‐inhibited BDNF production in hippocampal neurons, and accordingly we focused on the mechanism responsible for the beneficial effect of 25 μM propofol.

### 3.3 The beneficial effect of propofol on BDNF expression was mediated through regulating the phosphorylation of ERK and CREB

**FIGURE 3:**
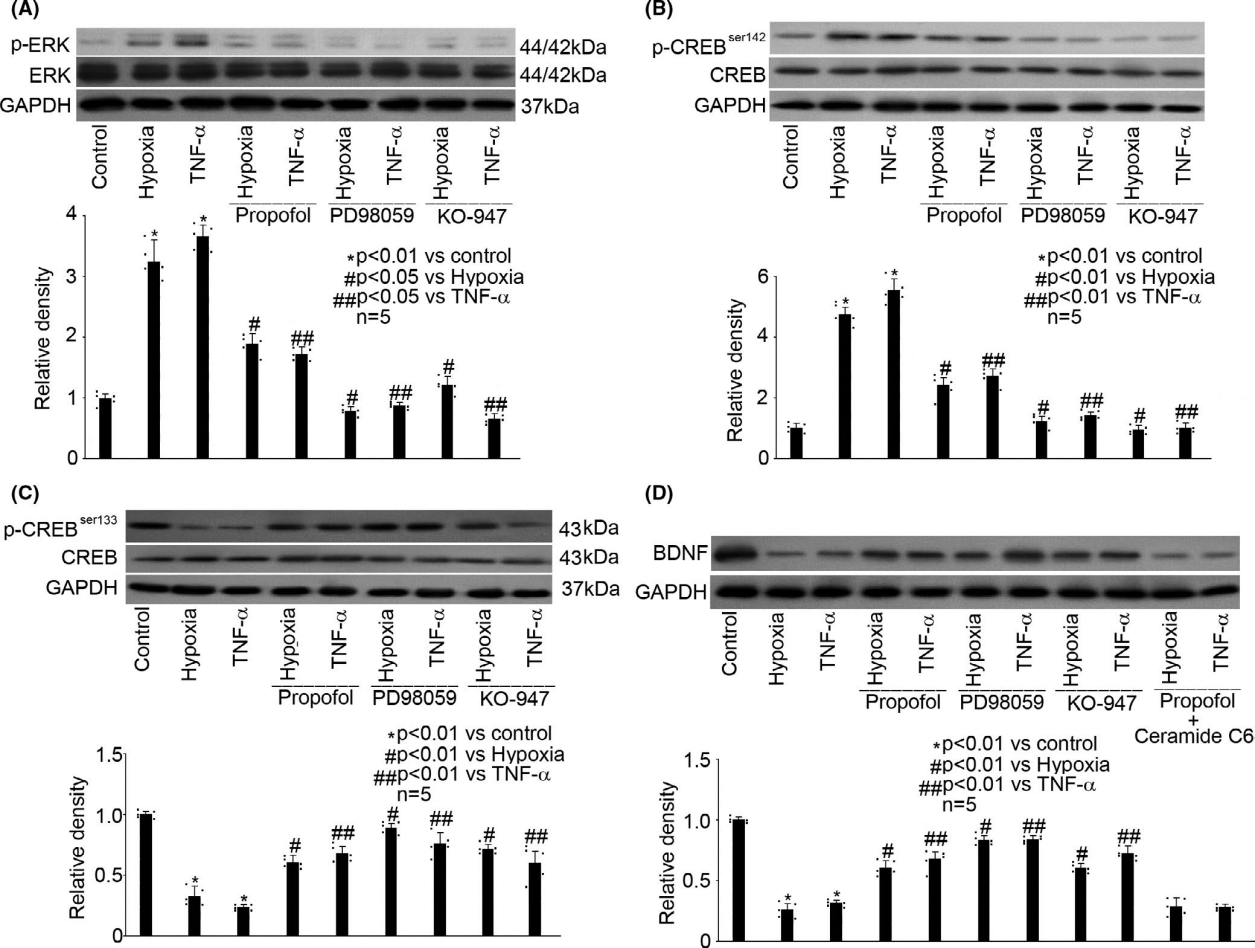
The beneficial effect of propofol on BDNF expression was mediated through regulating the phosphorylation of ERK and CREB. (A) In hippocampal neurons, hypoxia (5% O_2_, 3h) and TNF‐α (40 ng/mL, 3h) increased the phosphorylation of ERK, which was attenuated by 25 μM propofol, 10 μM PD98059, or 10 μM KO‐947. The upper panel was a representative experiment and the lower panel was the summary of densitometric data from five separate experiments. GAPDH served as loading control. Data were expressed as normalized ratio of protein band density of phosphorylated ERK against ERK, which was normalized with GAPDH, and were presented as mean ± standard deviation. (B) In hippocampal neurons, hypoxia (5% O_2_, 3h) and TNF‐α (40 ng/mL, 3h) increased the phosphorylation of CREB at Ser142 (p‐CREB ^Ser142^), which was reversed by 25μM propofol, 10μM PD98059, or 10μM KO‐947. (C) In hippocampal neurons, hypoxia (5% O_2_, 3h) and TNF‐α (40 ng/mL, 3h) reduced the phosphorylation of CREB at Ser133 (p‐CREB ^Ser133^), which was reversed by 25μM propofol, 10μM PD98059, or 10μM KO‐947. (D) In hippocampal neurons, hypoxia and TNF‐α reduced BDNF expression, which was reversed by 25μM propofol, 10μM PD98059, or 10μM KO‐947, and the beneficial effect of propofol on BDNF production was abolished by 10μM Ceramide C6

We revealed that in rat hippocampal neurons, hypoxia (5% O_2_, 3h) and TNF‐α (40 ng/mL, 3h) increased the phosphorylation of ERK, which was attenuated by 25 μM propofol, 10 μM PD98059 (a selective ERK inhibitor), or 10 μM KO‐947 (a potent and specific ERK inhibitor) (Figure 3A). We also detected that hypoxia (5% O_2_, 3h) and TNF‐α (40 ng/mL, 3h) increased the phosphorylation of CREB at Ser142 (p‐CREB^Ser142^) while reduced the phosphorylation of CREB at Ser133 (p‐CREB^Ser133^), which were both reversed by 25 μM propofol, 10 μM PD98059, or 10 μM KO‐947 (Figure 3B,C). Consistently, we demonstrated that 10 μM PD98059 and 10 μM KO‐947 could attenuate the inhibitory effect of hypoxia and TNF‐α on BDNF expression, which is similar to the effect of propofol (Figure 3D). In addition, we reported that the beneficial effect of propofol on hypoxia‐ and TNF‐α‐inhibited BDNF expression was abolished by the presence of 10 μM ERK activator (Ceramide C6) (Figure 3D).

### 3.4 Hypoxia and TNF‐α had no effect on TrkB expression, truncation, or phosphorylation in rat hippocampal neurons and astrocytes

**FIGURE 4:**
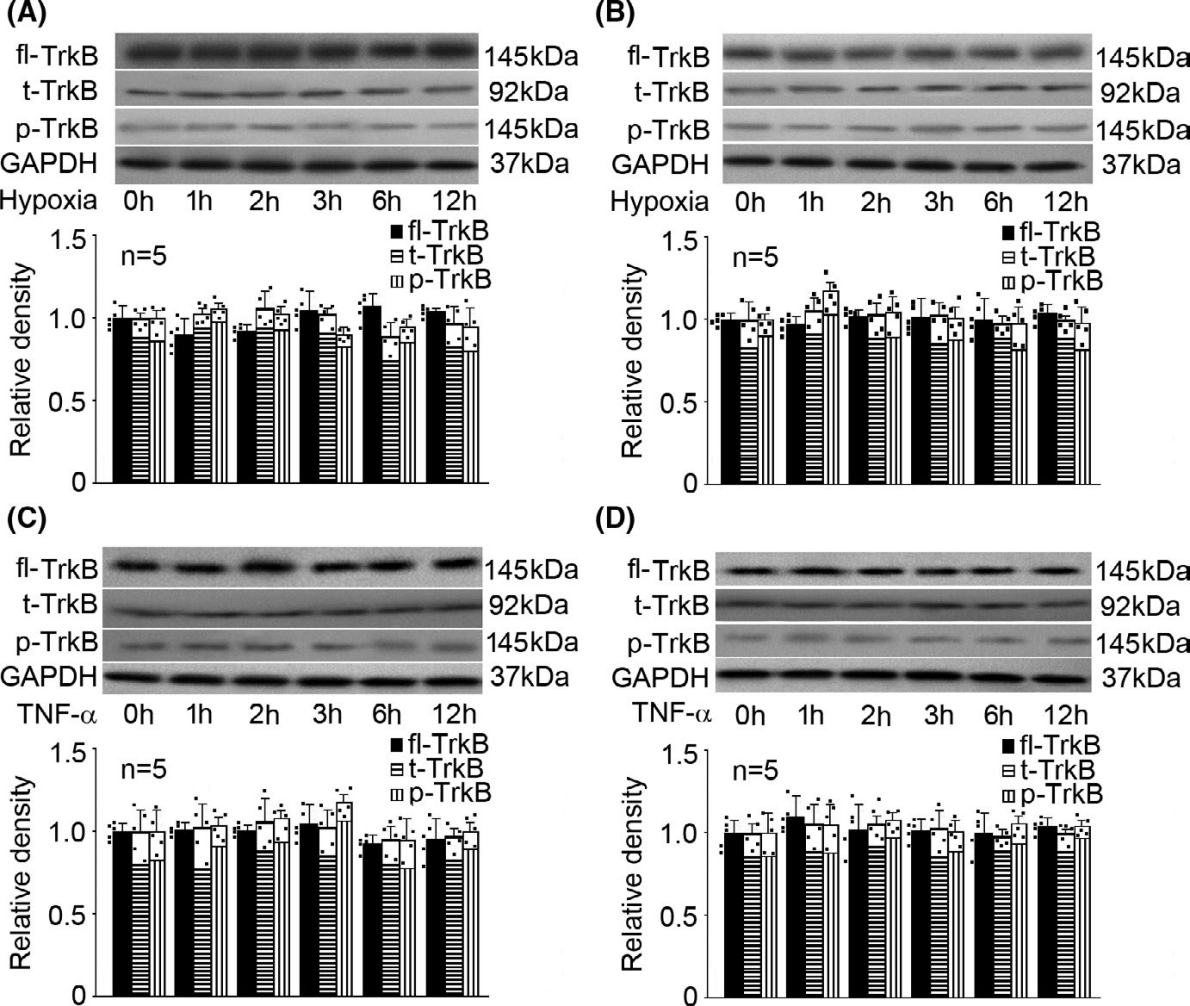
Hypoxia and TNF‐α had no effect on full‐length TrkB (fl‐TrkB) expression, truncation (t‐TrkB), or phosphorylation (p‐TrkB) in rat hippocampal neurons and astrocytes. The upper panel was a representative experiment and the lower panel was the summary of densitometric data from five separate experiments. GAPDH served as loading control. Data were expressed as normalized ratio of protein band density of p‐TrkB or t‐TrkB against fl‐TrkB, which was normalized with GAPDH, and were presented as mean ± standard deviation. (A) In rat hippocampal neurons, hypoxia had no effect on the expression, truncation, or phosphorylation of TrkB. (B) In astrocytes, hypoxia had no effect on the expression, truncation, or phosphorylation of TrkB. (C) In rat hippocampal neurons, TNF‐α had no effect on the expression, truncation, or phosphorylation of TrkB. (D) In astrocytes, TNF‐α had no effect on the expression, truncation, or phosphorylation of TrkB

Rat hippocampal neurons and astrocytes were cultured and exposed to hypoxia (5% O_2_) or TNF‐α (40 ng/mL) treatment for different duration (0, 1, 2, 3, 6, and 12h), and the expression, truncation, as well as phosphorylation of TrkB were measured. As shown in Figure 4, we reported that hypoxia had no effect on the expression, truncation, or phosphorylation of TrkB in rat hippocampal neurons (Figure 4A) and in astrocytes (Figure 4B). Also, TNF‐α had no effect on the expression, truncation, or phosphorylation of TrkB in rat hippocampal neurons (Figure 4C) and astrocytes (Figure 4D).

### 3.5 Propofol induced TrkB phosphorylation in rat hippocampal neurons

**FIGURE 5:**
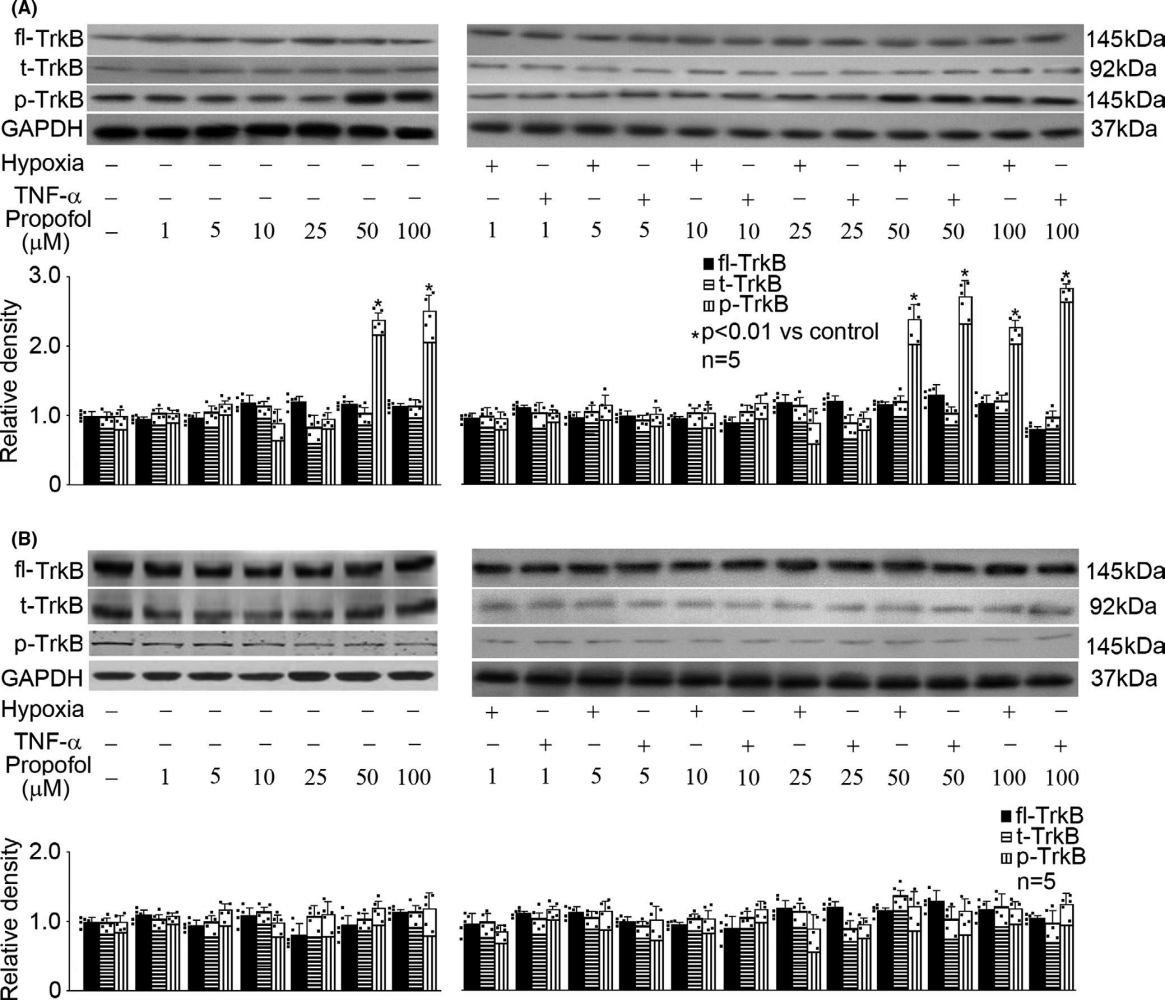
Propofol induced TrkB phosphorylation in hippocampal neurons. The upper panel was a representative experiment and the lower panel was the summary of densitometric data from five separate experiments. GAPDH served as loading control. Data were expressed as normalized ratio of protein band density of p‐TrkB or t‐TrkB against fl‐TrkB, which was normalized with GAPDH, and were presented as mean ± standard deviation. (A) In hippocampal neurons, propofol had no effect on TrkB expression or truncation, but induced TrkB phosphorylation. (B) In astrocytes, propofol had no effect on TrkB expression, truncation, or phosphorylation (B)

We treated rat hippocampal neurons and astrocytes with different concentrations of propofol (1, 5, 10, 25, 50, and 100 μM) for 1h, followed by hypoxia (5% O_2_, 3h) or TNF‐α (40 ng/mL, 3h) treatment, and examined the expression, truncation, and phosphorylation of TrkB. We noticed that in rat hippocampal neurons, propofol had no effect on TrkB expression or truncation (Figure 5A). Interestingly, propofol (50 and 100 μM) induced TrkB phosphorylation, regardless of whether cells were exposed to hypoxia, TNF‐α, or not (Figure 5A, *P* < 0.05 vs. control). However, propofol had no effect on TrkB expression, truncation or phosphorylation in astrocytes (Figure 5B). Thereafter, we intended to investigate the mechanism responsible for 50 μM propofol‐induced TrkB phosphorylation in hippocampal neurons.

### 3.6 Propofol‐induced TrkB phosphorylation was carried out via modulating p35 expression and Cdk5 activation

**FIGURE 6:**
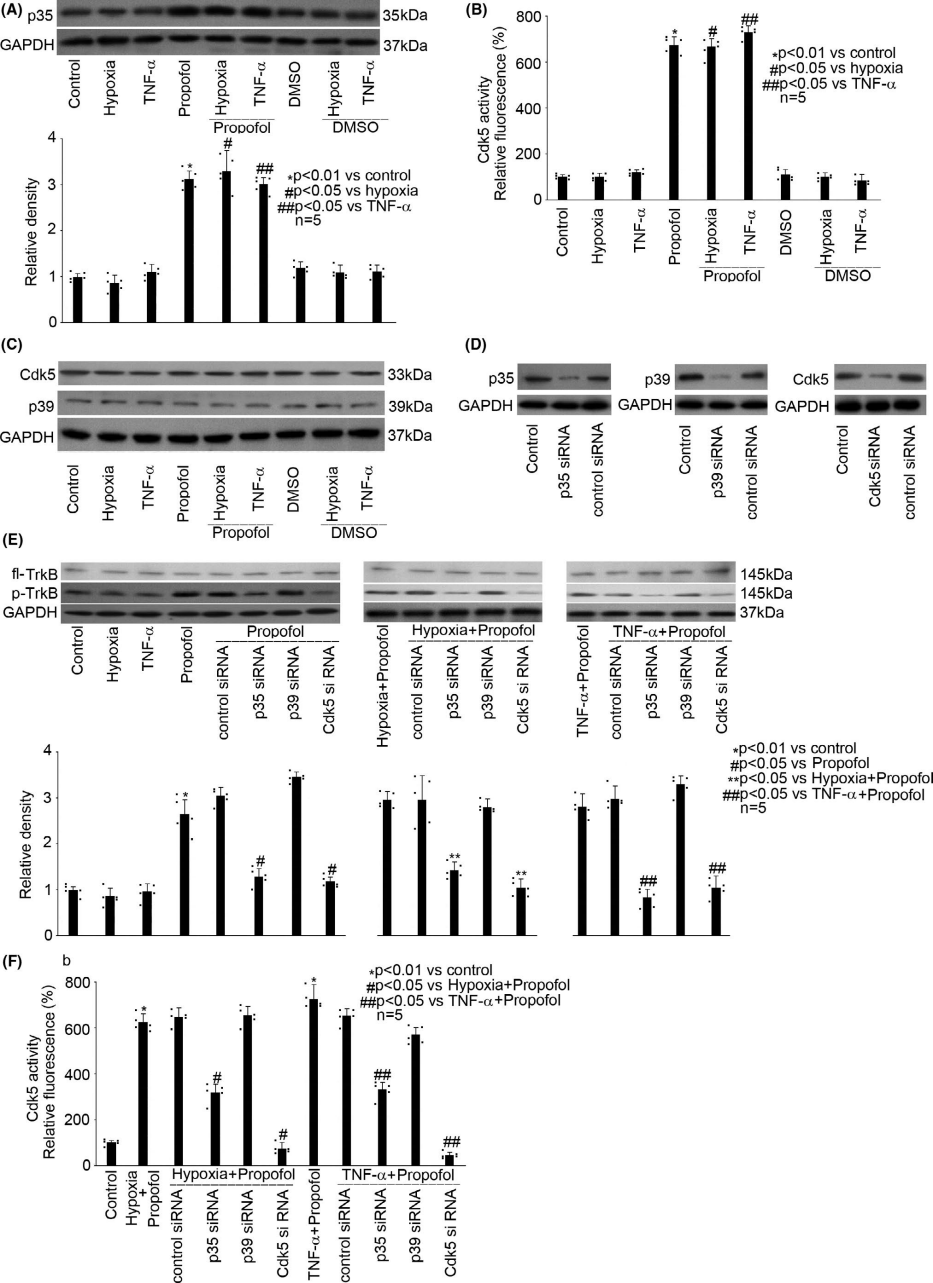
Propofol‐induced TrkB phosphorylation was carried out via modulating p35 expression and Cdk5 activation in hippocampal neurons. (A) Propofol induced the expression of p35. The upper panel was a representative experiment and the lower panel was the summary of densitometric data from five separate experiments. GAPDH served as loading control. Data were expressed as normalized ratio of protein band density of p35 against GAPDH, and were presented as mean ± standard deviation. (B) Propofol induced the activation of Cdk5. Data were expressed as relative fluorescence compared with that of untreated control cells, and were presented as mean ± standard deviation. One hundred percent activity was set for control cells. (C) Propofol had no effect on the expression of Cdk5 and p39. The panel was a representative experiment, and GAPDH served as loading control. (D) Transfection efficiency of siRNAs against p35, p39, and Cdk5 was evaluated by Western blot. Untransfected neurons served as normal control, and control siRNA‐transfected neurons served as transfection control. The panel was a representative experiment. (E) Transfection of siRNA against p35 or Cdk5 alleviated propofol‐induced TrkB phosphorylation, while transfection of siRNA against p39 had no such effect. The upper panel was a representative experiment and the lower panel was the summary of densitometric data from five separate experiments. GAPDH served as loading control. Data were expressed as normalized ratio of protein band density of p‐TrkB against fl‐TrkB, which was normalized with GAPDH, and were presented as mean ± standard deviation. (F) Blockade of p35 and Cdk5 alleviated Cdk5 activation. Data were expressed as relative fluorescence compared with that of untreated control cells, and were presented as mean ± standard deviation. One hundred percent activity was set for control cells

We found that in hippocampal neurons, hypoxia (5% O_2_, 3h) and TNF‐α (40 ng/mL, 3h) did not affect p35 expression, while 50μM propofol, rather than 0.1% DMSO, induced the expression of p35 regardless of the exposure to hypoxia or TNF‐α (Figure 6A). Consistently, although hypoxia and TNF‐α had no effect on the activation of Cdk5 (Figure 6B), it was activated by 50 μM propofol but not 0.1% DMSO. In addition, hypoxia, TNF‐α, propofol, and DMSO had no effect on the expression of Cdk5 and p39 (Figure 6C). Then, we applied siRNA technology to confirm the involvement of p35 and Cdk5 in propofol‐mediated TrkB phosphorylation. As shown in Figure 6D, we demonstrated that the siRNA targeting p35, p39, and Cdk5 could effectively diminish the expression of p35, p39, and Cdk5, respectively. More importantly, we revealed that blockade of p35 and Cdk5 alleviated propofol‐induced TrkB phosphorylation, while blockade of p39 had no such effect (Figure 6E). To confirm the relationship between p35 and Cdk5, we blocked p35 expression by siRNA technology and examined Cdk5 activity. As shown in Figure 6F, we revealed that the propofol‐induced Cdk5 activation was alleviated by blockade of p35 and Cdk5, rather than p39 expression (Figure 6F).

## 4 DISCUSSION

### 4.1 Hypoxia‐ and TNF‐α‐mediated dysregulation of BDNF/TrkB pathway

BDNF belongs to the neurotrophin (NT) family, which is composed of four structurally related members: BDNF, neuronal growth factor (NGF), neurotrophin‐3 (NT‐3), and NT‐4/5.^28^ It has been well recognized that BDNF is the most abundant endogenous neurotrophic factor in the body, and reduced levels of BDNF were reported to play a key role in rodent models during the development of neurological disorders, such as cerebral ischemia‐reperfusion injury^2^ and neuroinflammation‐related brain injury.^3^ Besides, it is clear that the NT actions are mediated by interacting with two transmembrane receptors with different affinity. Generally, all members of the NT family, especially their precursors bind to p75NTR with low affinity, whereas mature NTs bind to different Trk receptors, including TrkA, TrkB, and TrkC, with high affinity according to ligand selectivity. TrkA has been identified as the preferred receptor for NGF, TrkB for BDNF, and TrkC for NT‐3/4/5.^29^ Prior findings highlighted the role of p75NTR in neuronal pathological conditions such as traumatic brain injury, intracranial hemorrhage, ischemic/hypoxia stroke, and neurological diseases, by modulating neuronal apoptosis, axonal elongation and degeneration, myelination, cell proliferation, and synaptic plasticity.^30^ In contrast, Trk receptors could activate signaling pathways that are important for cellular activities, including cell proliferation, survival, and axon, as well as dendrite outgrowth.^31^ In the current in vitro study, we mainly examined matured form of BDNF, and accordingly, we only focused on TrkB receptor. After BDNF binding, TrkB undergoes dimerization, followed by phosphorylation of intracellular tyrosine kinase residues which act as docking sites for adaptor proteins that recruit additional kinases, leading to activation of intracellular signaling pathways. The activation of BDNF/TrkB is required for neuron differentiation, survival, synaptic plasticity, and neurotransmitter regulation, while dysregulation of BDNF/TrkB contributes many pathological processes, including traumatic brain injury, brain ischemic injury, and neurodegenerative diseases.^13^

It is known that BDNF/TrkB dysregulation was correlated with several harmful insults, such as oxidative stress and inflammation.^32^ In the current study, we focused on two factors (hypoxia and inflammation), which are major players in the pathogenesis of neurological disorders, and two cell types (hippocampal neurons and astrocytes), which are major sources of BDNF in CNS. We found that both hypoxia and inflammation reduced the expression of BDNF in hippocampal neurons and astrocytes (Figure 1). However, they had no effect on TrkB expression, truncation, or phosphorylation (Figure 4). Since we only focused the role of mature BDNF in this study, we did not examine p75NTR, TrkA, or TrkC. In addition, it is known that TrkB has two isoforms: truncated TrkB (TrkB‐TC) and full‐length TrkB (TrkB‐FL). TrkB‐TC may act as negative modulators of TrkB‐FL. A previous study showed that excitotoxic stimulation of cultured rat hippocampal neurons with glutamate down‐regulated TrkB‐FL while up‐regulated TrkB‐TC, which resulted in dysregulation of BDNF/TrkB signaling.^33^ Nevertheless, we found neither hypoxia nor TNF‐α affected the truncation of TrkB (Figure 4). Interestingly, our findings differ from a previous animal study that reported an increase in BDNF and TrkB expression in chronic cerebral ischemia in the hippocampus of aged rats.^24^ We postulated that the discrepancy could be due to two reasons: firstly, we examined acute hypoxia and inflammation rather than chronic ischemia; secondly, our study was carried out in neurons rather than in aged animals. We concluded that in hippocampal neurons and astrocytes, hypoxia and inflammation may cause dysregulation of BDNF/TrkB pathway mainly through modulation of BDNF expression. Our data indicated that hippocampal neurons and astrocytes responded differently to hypoxia and TNF‐α. In other words, in different cells, the significant effects of hypoxia/TNF‐α on BDNF expression appeared at different time points and reached different magnitude. This could be explained by the phenotypes of cells. On the other hand, different intracellular signaling pathway may be involved. Significant effects appeared mostly after 3h treatment which correlated with cell viability, suggesting a potential role, at least in part, of the reduced cell viability in reduced BDNF expression.

### 4.2 The protective property of propofol against hypoxia‐ and TNF‐α‐mediated BDNF/TrkB dysregulation

Propofol is an intravenous anesthetic widely used in clinical anesthesia and sedation. In addition, it has a variety of biological effects on the protection of organs, including brains,^34^ hearts,^35^ and kidneys.^36^ Currently, the neuroprotective property of propofol in the CNS and the underlying mechanism are of great interests. A large amount of in vitro studies revealed that propofol may improve BBB function,^21^ protect neuron apoptosis^18^ and autophagy,^17^ and maintain microglia function.^37^ In addition, animal studies demonstrated that propofol may improve brain function in rats with ischemia‐reperfusion injury^38^ and may ameliorate neuroinflammatory injury in rats.^39^, ^40^

Recently, the role of BDNF/TrkB signaling in the neuroprotective property of propofol gains interests. An animal study indicated that propofol may protect chronic ischemic cerebral injury in aged rats via modulating BDNF/TrkB pathway.^24^ In that animal study, it was reported that low‐dose propofol (10 mg/kg, intraperitoneally) promoted the expression of BDNF and TrkB, but high‐dose propofol (50 mg/kg, intraperitoneally) inhibited their expression. Consistently, our *in vitro* study demonstrated that 25–50 μM propofol induced BDNF expression in hippocampal neurons which were exposed to hypoxia or TNF‐α (Figure 2). Meanwhile, we found propofol had no effect on TrkB expression, while increased its phosphorylation regardless of whether the hippocampal neurons were exposed to hypoxia/TNF‐α or not (Figure 5). We postulated that the difference in the amount of propofol administration and the difference in experiment model may account for the discrepancy. In contrast, our data implied that astrocytes may not be a target for propofol with regard to BDNF/TrkB dysregulation (Figures 2 and 5). We note that the beneficial concentration of propofol was 25–50 μM, which is within the plasma range of propofol during general anesthesia and is clinically relevant. Accordingly, we concluded that propofol may regulate hypoxia‐ and TNF‐α‐mediated BDNF/TrkB dysregulation, through BDNF expression and TrkB phosphorylation in hippocampal neurons, but not in astrocytes.

### 4.3 ERK/CREB and p35/Cdk5 were involved in the beneficial effect of propofol against hypoxia‐ and TNF‐α‐mediated BDNF/TrkB dysregulation

The mechanisms involved in the neuroprotective effect of propofol against hypoxia‐ and inflammation‐mediated injuries have been widely studied both in the in vitro model and in the animal model, and the potential mechanisms may include but not be limited to phosphatidylinositol‐3‐kinase/protein kinase B (PI3K/PKB) pathway,^38^ PIM‐1/nitric oxide synthase (NOS)/NO pathway,^41^ rapamycin/ribosomal protein S6 kinase beta‐1 pathway,^42^ Janus kinase/signal transducer and activator of transcription (JAK/STAT) pathway,^43^ HSF1/heat shock protein 27 (HSP27) and Nrf2/ HSP32 pathway,^22^ and Ca^2+^/calmodulin‐dependent protein kinase II (CAMKII)/extracellular regulated protein kinases (ERK)/NF‐κB pathway.^21^, ^23^ However, the molecular mechanism responsible for propofol‐modulated BDNF/TrkB regulation still remains unknown.

Here, in the present study, our data suggested that ERK/CREB signaling is involved in hypoxia‐ and TNF‐α‐mediated BDNF/TrkB dysregulation (Figure 3). More specifically, we believed that ERK/CREB signaling plays a key role in the beneficial effect of propofol on BDNF production. The pivotal role of ERK/CREB in BDNF production has previously been shown in the brain of mice^44^ and rats.^45^ It is well known that CREB could be phosphorylated by protein kinases such as protein kinase A (PKA), protein kinase C (PKC), PI3K, CAMKII, and ERK at different site such as Ser133 and Ser142, and it is recognized that most kinases induce p‐CREB^Ser133^, which increases CREB transcriptional activity, while some kinases induce p‐CREB^Ser142^, which decreases its activity. Although p‐CREB^Ser133^ has already been shown to be correlated with BDNF production in rat model^46^ and in rat cortical neurons^47^ as well as in mouse hippocampal neurons,^48^ the role of p‐CREB^Ser142^ has rarely been investigated. Given our data showing that ERK inhibitor attenuated hypoxia‐ and TNF‐α‐mediated phosphorylation of CREB^Ser142^ and CREB^Ser133^, and that the presence of ERK activator markedly abolished the effects of propofol on CREB phosphorylation (Figure 3), it is likely that ERK functions upstream of CREB. While our data did not prove a definitive role of CREB in BDNF production, our finding of correlation between the phosphorylation of CREB and propofol‐induced BDNF production is a novel aspect of the current study, particularly the finding of an increase in p‐CREB^Ser133^ and a decrease in p‐CREB^Ser142^.

In addition, our data implicated a role of p35/Cdk5 in propofol‐modulated BDNF/TrkB pathway, especially on TrkB, based on our data showing that blockade of p35/Cdk5 abolished the effect of propofol on TrkB phosphorylation (Figure 5). Cdk5 is a small serine/threonine kinase abundant in postmitotic neurons, and the activation of Cdk5 requires the binding of one of its two specific activators, p35 or p39, in the developing cerebral cortex and hippocampus.^49^, ^50^ It is known that p35 and p39 share approximately 60% sequence homology and exhibit differential developmental expression in the brain. The expression of p35 protein is high throughout the embryonic stage, whereas p39 expression increases during postnatal differentiation. Although *in vitro* experiments suggested that p35 and p39 share similar substrate specificity, they are spatially segregated within neurons and have different biochemical properties.^51^ Previous study indicated that p35/Cdk5‐mediated phosphorylation of target protein is required for hypoxia‐induced xanthine oxidoreductase hyperactivation in the lung,^52^ and p35/Cdk5 has been shown to be responsible for phosphorylation of TrkB, neurofilament proteins, and tau protein in brain.^50^, ^53^ Consistently, we found p35, rather than p39, is critical for TrkB phosphorylation in the hippocampal neurons that were exposed to hypoxia, TNF‐α, and propofol (Figure 6E). Meanwhile, based on the findings that blockade of p35 expression attenuated Cdk5 activity (Figure 6F), we concluded that p35 functions upstream of Cdk5.

In the present in vitro study, the beneficial concentration of propofol was 25 and 50 μM, which is about 4.5 and 9 μg/mL. In clinical practice, during general anesthesia with propofol, plasma concentration was usually kept at 2–6 μg/mL. It seems 50 μM is beyond physiological concentration range, and may not be achieved during general anesthesia. However, given the fact that in vitro system is different from in vivo system and that the concentration of an agent used in in vitro study maybe 10 times higher than that used in clinical practice, we believe that the protective concentration found in this study is clinically relevant. On the other hand, although this is an in vitro study carried out in hippocampal neurons, it is of great clinical relevance and significance. Our findings may provide rationale for choosing propofol as an intraoperative or sedative agent in those patients who are under the risk of cerebral ischemia or inflammation, and this need to be confirmed by following animal study.

### 4.4 Limitation

Our study has several limitations. Firstly, we focused on ERK/CREB and p35/Cdk5 in hypoxia‐ and TNF‐α‐ as well as propofol‐mediated regulation of BDNF/TrkB pathway, without investigating detailed analysis of other components in the signaling pathway. Secondly, it is known that p‐CREB may be dephosphorylated by phosphatase PP1 and PP2A to keep the balance of its phosphorylation status. However, in the study, we did not examine the effect of hypoxia, TNF‐α, or propofol on the expression and activity of these enzymes.

## 5 CONCLUSION

In this *in vitro* study, we reported that in rat hippocampal neurons, propofol via modulating ERK/CREB signaling pathway may reverse hypoxia‐ and TNF‐α‐mediated reduction in BDNF. In addition, we demonstrated that in hippocampal neurons, propofol via activating p35/Cdk5 pathway induces TrkB phosphorylation. Taken together, our findings suggested a novel protective effect and mechanism of propofol against hypoxia‐ and TNF‐α‐induced malfunction of hippocampal neurons.

## CONFLICT OF INTEREST

The authors confirm that there are no conflicts of interest.

## AUTHOR CONTRIBUTIONs

Weiping Tao performed research and wrote the paper; Xuesong Zhang performed research and wrote the paper; Juan Ding performed research and analyzed data; Shijian Yu performed research; Peiqing Ge analyzed data; Jingfeng Han analyzed data; Xing Luo analyzed data and wrote the paper; Wei Cui designed research and revised the paper; Jiawei Chen designed research and revised as well as approved the paper.

## Supporting information

Fig S1

Fig S2

## Data Availability

The data that support the findings of this study are available from the corresponding author upon reasonable request.
