# Supplementary figures and images for: The effect of propofol on hypoxia‐ and TNF‐α‐mediated BDNF/TrkB pathway dysregulation in primary rat hippocampal neurons
Source: CNS Neurosci Ther. 2022 Feb 3;28(5):761–74. doi: 10.1111/cns.13809 (PMC8981449; doi:10.1111/cns.13809)

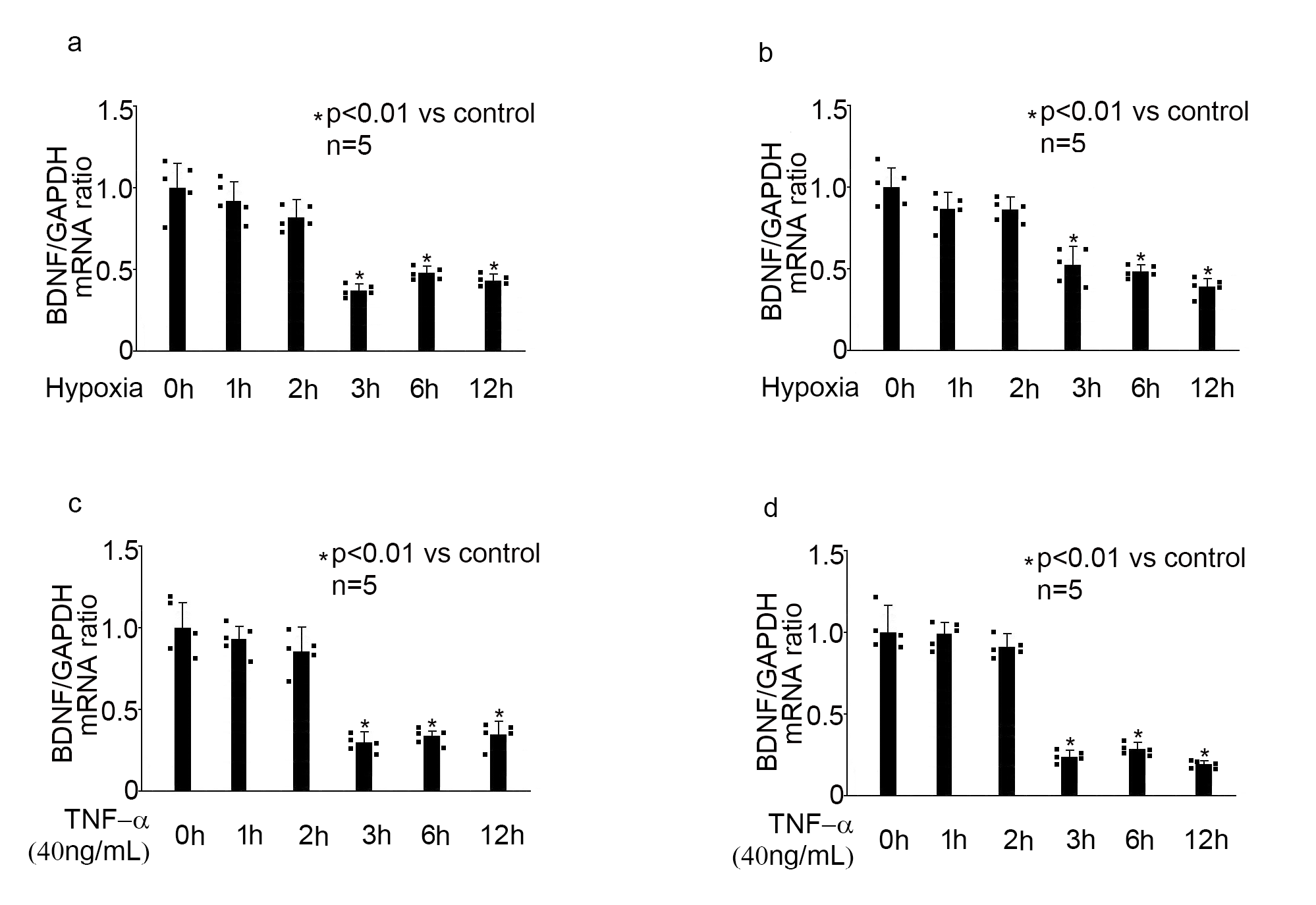

Supplement: Supplementary file 1 — Fig S1 [file CNS-28-761-s002.tif]

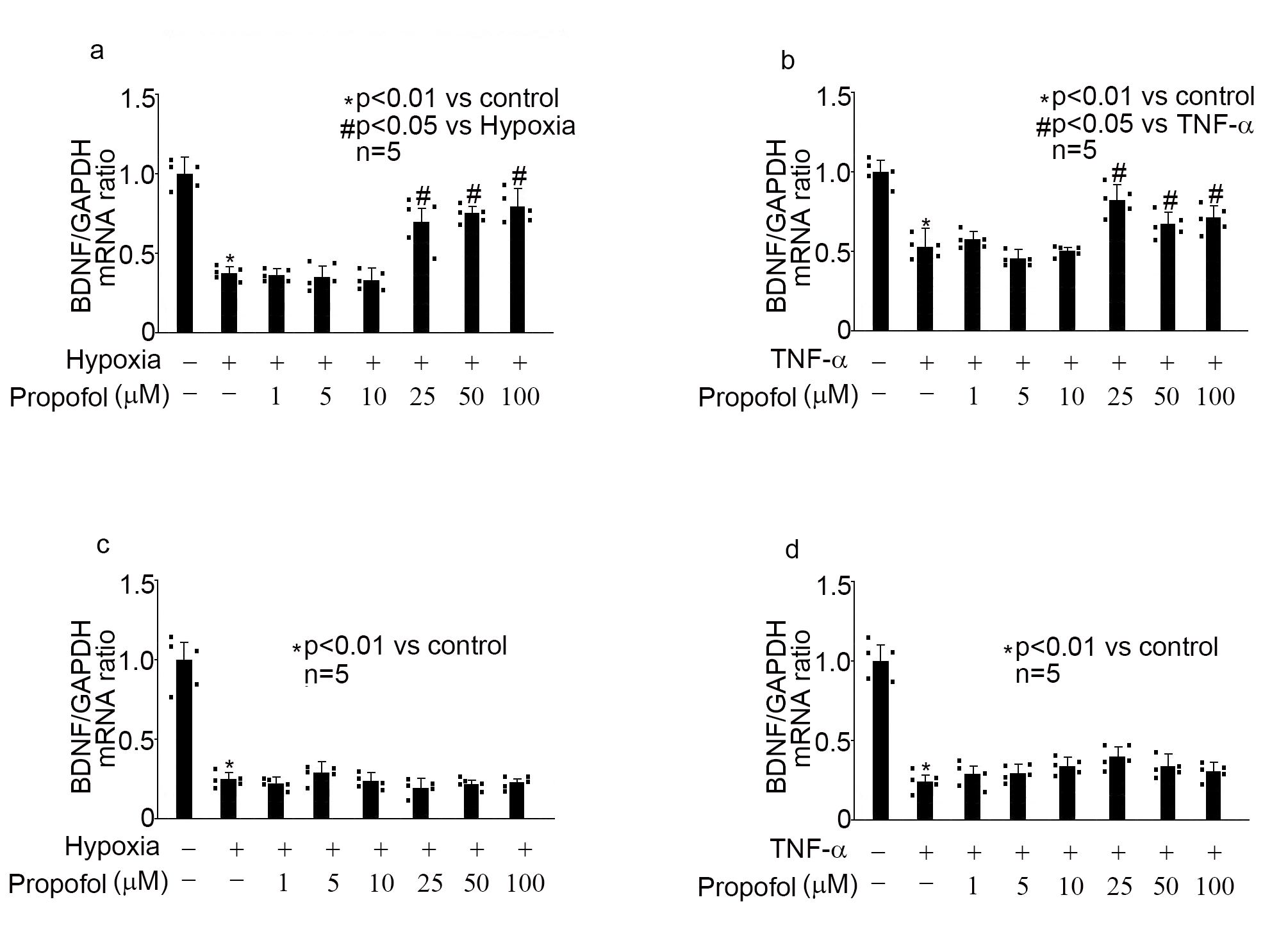

Supplement: Supplementary file 2 — Fig S2 [file CNS-28-761-s001.tif]
